# Laparoscopy versus open appendectomy for elderly patients, a meta-analysis and systematic review

**DOI:** 10.1186/s12893-019-0515-7

**Published:** 2019-05-28

**Authors:** Dayun Wang, Ting Dong, Yue Shao, Tingting Gu, You Xu, Yuan Jiang

**Affiliations:** Department of Operating Room, Yijishan Hospital of Wannan Medical College, No. 2, Zheshan West Road, Wuhu City, 241001 Anhui Province China

**Keywords:** Appendicitis, Elderly population, Laparoscopy, Open appendectomy, Meta-analysis

## Abstract

**Background:**

Appendicitis in elderly patients is associated with increased risk of postoperative complications. The choice between laparoscopy and open appendectomy remains controversial in treating elderly patients with appendicitis.

**Methods:**

Comprehensive search of literature of MEDLINE, Embase, Cochrane Library and ClinicalTrials was done in January 2019. Studies compared laparoscopy and open appendectomy for elderly patients with appendicitis were screened and selected. Postoperative mortality, complications, wound infection, intra-abdominal abscess and operating time, length of hospital stay were extracted and analyzed. The Review Manage 5.3 was used for data analysis.

**Results:**

Twelve studies with 126,237 patients in laparoscopy group and 213,201 patients in open group. Postoperative mortality was significantly lower following laparoscopy (OR, 0.33; 95% CI, 0.28 to 0.39). Postoperative complication and wound infection were reduced following laparoscopy ((OR, 0.65 95% CI, 0.62 to 0.67; OR,0.27, 95% CI, 0.22 to 0.32). Intra-abdominal abscess was similar between LA and OA (OR,0.44;95% CI, 0.19 to 1.03). Duration of surgery was longer following laparoscopy and length of hospital stay was shorter following laparoscopy (MD, 7.25, 95% CI, 3.13 to 11.36; MD,-2.72, 95% CI,-3.31 to − 2.13).

**Conclusions:**

Not only laparoscopy is safe and feasible, but also it is related with decreased rates of mortality, post-operative morbidity and shorter hospitalization.

**Electronic supplementary material:**

The online version of this article (10.1186/s12893-019-0515-7) contains supplementary material, which is available to authorized users.

## Background

Appendicitis is the most common cause of abdominal pain and a prevalent reason for emergency surgery. The risks of developing appendicitis through lifetime is approximately 8.6% for male and 6.7% for female [1]. Aging of population has been a serious problem in many counties, according to prediction, by 2050, the population of elderly people (age more than 65) will be around 498 million in China [2]. The prevalence of appendicitis will increase following the population changes [3]. Previous studies demonstrated that appendicitis in elderly are associated with higher risk of perforation and complications due to more comorbidities and more challenge of accurate diagnosis [4, 5]. Therefore, precise diagnosis coupled with appropriate procedure are crucial for treating appendicitis in elderly population [6, 7].

Laparoscopic appendectomy (LA) was first mentioned by Kurt Semm in 1983 [8], since that, numerous studies have focused on the comparison of laparoscopy and conventional open appendectomy (OA). In adults, LA is associated with less postoperative pain, faster recovery and less surgical complications. However, there are a great amount of debate concerning postoperative intra-abdominal abscess (IAA) after LA. A recent Cochrane review has demonstrated increased risk of IAA following LA, on the other hand, a cumulative meta-analysis by Ukai et al. demonstrated that increased risk of IAA following LA disappeared in studies published after 2001 [9–11]. The use of LA in elderly patients is still under debate. Previous studies suggested the same advantages of LA for elderly patients as for adults whereas some argued that the use of carbon dioxide for pneumoperitoneum increasing the risk of cardiovascular comorbidities.

In the present study, we searched several database for studies of LA versus OA for elderly patients and tried to reach a conclusion based on quantification analysis.

## Methods

The study was conducted following the published protocol for Systematic Reviews and Meta-Analyses [12].

### Search & Study selection

Comprehensive literature search of several database (MEDLINE, Embase, Cochrane Library, Clinical Trials) of relevant studies was conducted in Jan. 2019. The searching strategy was based on Mesh terms plus entry terms for each component of the PICOS question [13]. The exact searching terms were shown in Additional file 1. The reference lists of relevant studies were also screened. We also contacted the corresponding author for more information if necessary.

Selecting and screening of the studies were conducted by two independent reviewers (DY W and T D). When the two independent authors disagree with each other while screening, a call of term meeting was needed to discuss whether we should include the study or not.

### Eligibility criteria

Identified studies were collected for further selection if they meet the following eligibility criteria: comparison studies focused on laparoscopy and open appendectomy; enrolled patients older than 65 years old; complete records of clinical data and postoperative follow up records.

### Data collection & grading of individual study

The following data was extracted from the enrolled studies: name of the study, country, type of study design, baseline characteristic of the participants and outcomes. Assessing the quality of the study was based on the type of the study, for observational studies, we used the Newcastle Ottawa Quality assessment Scale [14] (NOS Scale).

### Data analysis

We performed data analysis by using Review Manager 5.3 Software. The choice between Fix or Random effects model was based on the degree of heterogeneity. The heterogeneity of the included studies was determined by I^2^ statistic. If I^2^ was greater than 70%, we used subgroup analysis to explore the cause of great heterogeneity.

The Peto odds ratio (OR) or Mean difference (MD) was calculated with 95% confidence intervals (CIs) for dichotomous outcomes and continuous outcomes. Funnel plot was conducted to detect publication bias for each pooled outcome.

A *P* value less than 0.05 was considered statistically significant.

## Results

### Study selection & characteristics

**Fig. 1:**
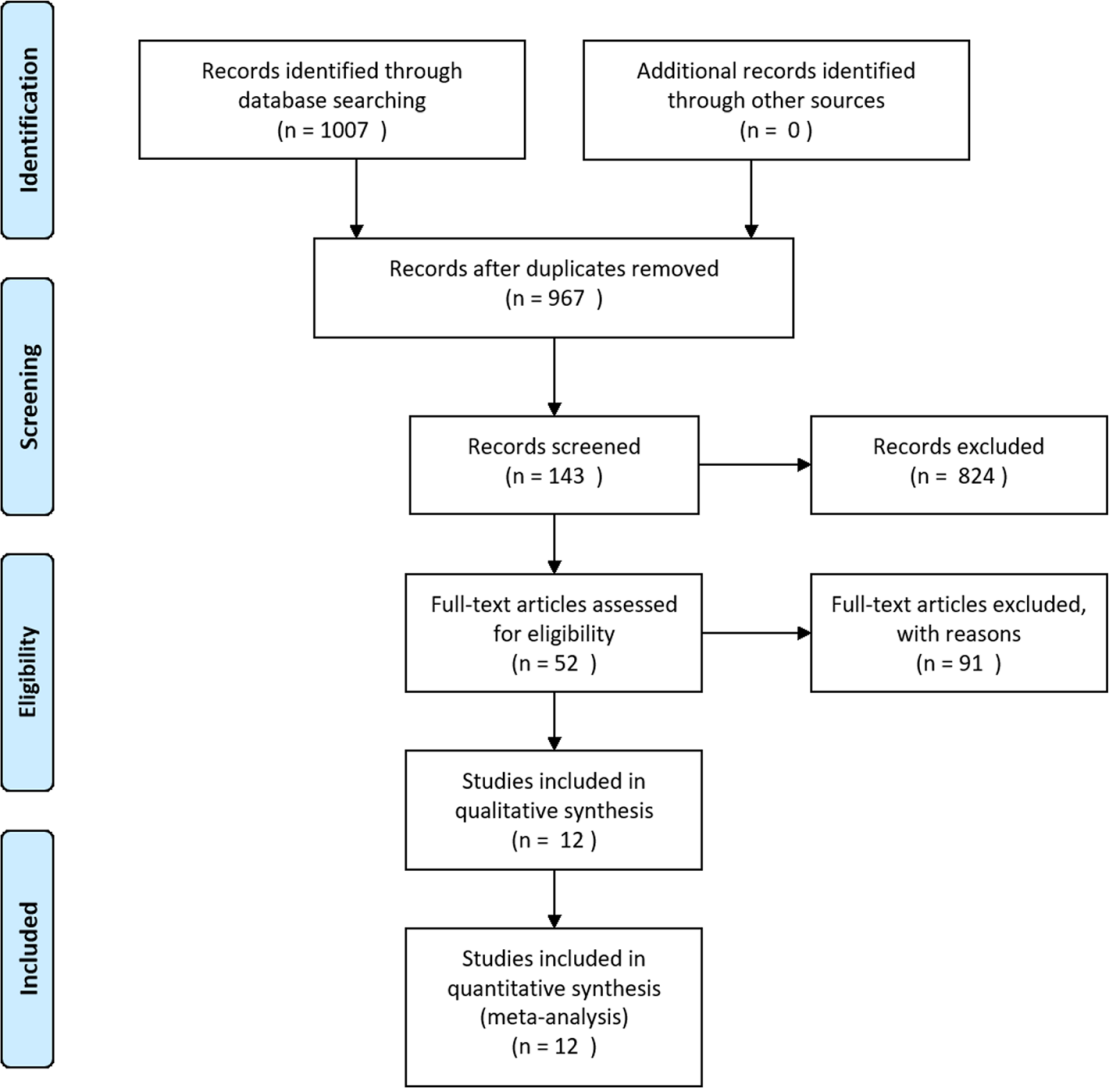
Searching and screening flow of included studies

The searching and screening process was shown in Fig. 1. Twelve studies were finally included [15–26]. The basic characteristics of the studies were summarized in Table 1. Two studies used the same database with potential overlap population [21, 23], however, after screening the full texts, we found that these studies reported different outcomes focusing on different aspect of the procedures, so we decided to include the two studies. All included studies were observational studies hence the NOS scale was used for assessment of quality. (Shown in Additional file 2). The quality score of studies varied from 7 to 9.

**Table 1:** Basic characteristics of the enrolled studies

	Year	Country	Design of study	No. of patients in LA group	No. of patients in OA group	Primary outcomes
Guller	2004	USA	Retrospective	1475	8001	LOS, Postoperative complications
Harrell	2006	USA	Retrospective	534	2188	LOS, Postoperative complications
Wang	2006	Taiwan, China	Retrospective	24	29	Duration of surgery, LOS
Paranjape	2007	USA	Retrospective	68	48	Duration of surgery, LOS, Complications
Kim	2011	USA	Retrospective	2235	1100	LOS, complications
Wu	2011	Taiwan, China	Retrospective	7.86	0.06	Duration of surgery, LOS, Complications
Masoomi	2012	USA	Retrospective	34,066	31,397	LOS, Complications
Farrerese	2013	Italy	Retrospective	19	20	Duration of surgery, LOS, Complications
Moazzez	2012	USA	Retrospective	2644	1030	Duration of surgery, LOS, Complications
Ward	2016	USA	Retrospective	87,209	170,276	Duration of surgery, LOS, Complications
Wu	2017	China	Retrospective	56	59	Duration of surgery, LOS, Complications
Yang	2017	China	Retrospective	80	65	Duration of surgery, LOS, Complications

*abbreviation: LOS* length of hospital stay

### Postoperative mortality

**Fig. 2:**
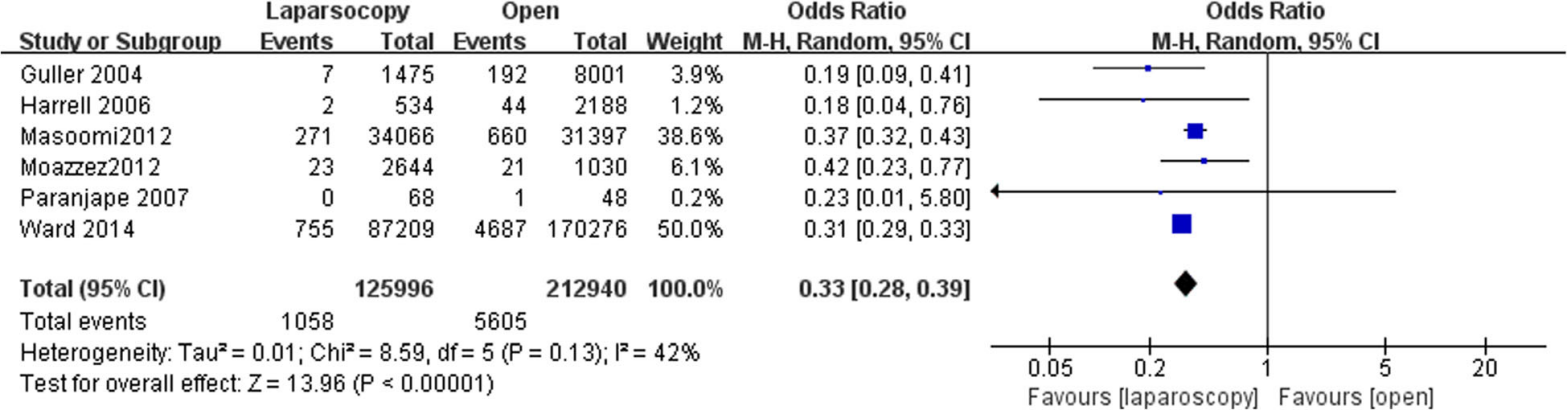
Postoperative mortality after laparoscopy and open appendectomy

**Fig. 3:**
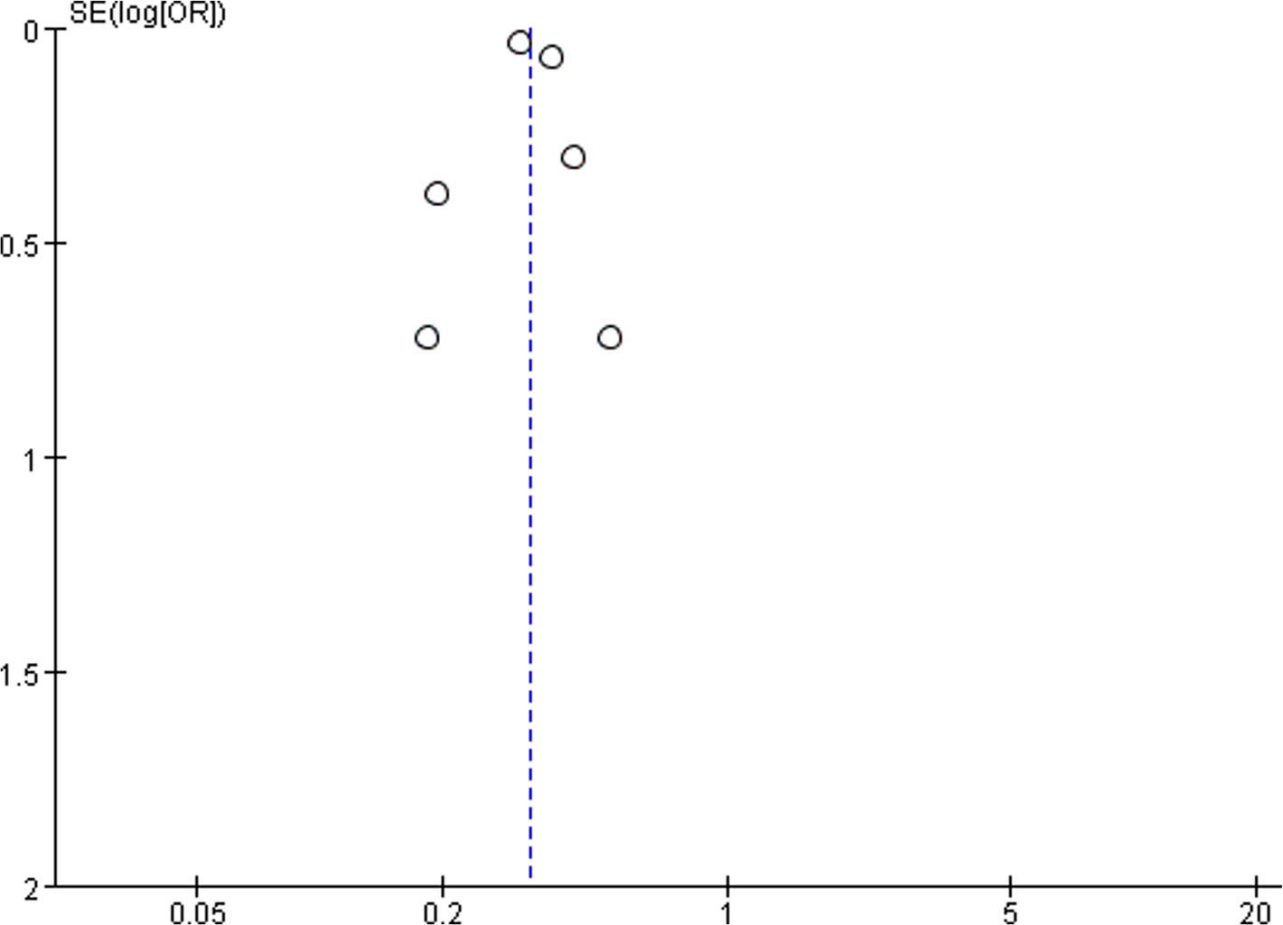
Funnel plot for postoperative mortality

Six studies reported postoperative mortality data with 125,996 in LA group and 212,940 patients in OA group. Postoperative mortality was significantly reduced following LA (OR, 0.33; 95% CI, 0.28 to 0.39, shown in Fig. 2). Moderate heterogeneity was found between the studies (I^2^ = 42%). Funnel plot for publication bias detection showed no obvious bias in postoperative mortality (Fig. 3).

### Overall complications

**Fig. 4:**
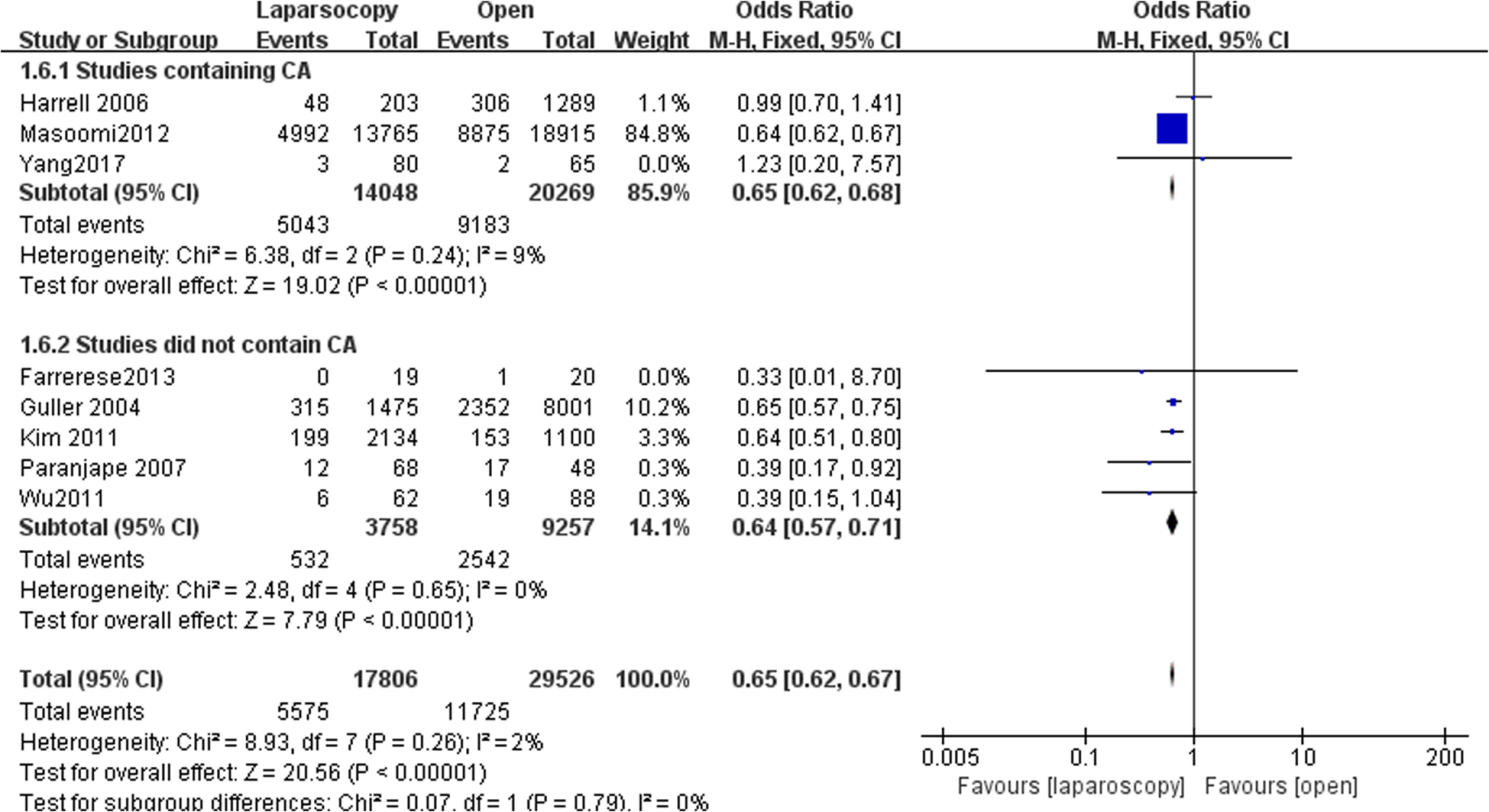
Subgroup analysis of postoperative complications after laparoscopy and open appendectomy

Eight studies reported overall complication data with 17,806 patients in LA group and 29,526 patients in OA group. Overall complication was significantly reduced following laparoscopy (OR, 0.65 95% CI, 0.62 to 0.67, shown in Fig. 4). We conducted subgroup analysis by dividing the studies into studies containing complicated appendicitis (CA) and studies which did not.

**Fig. 5:**
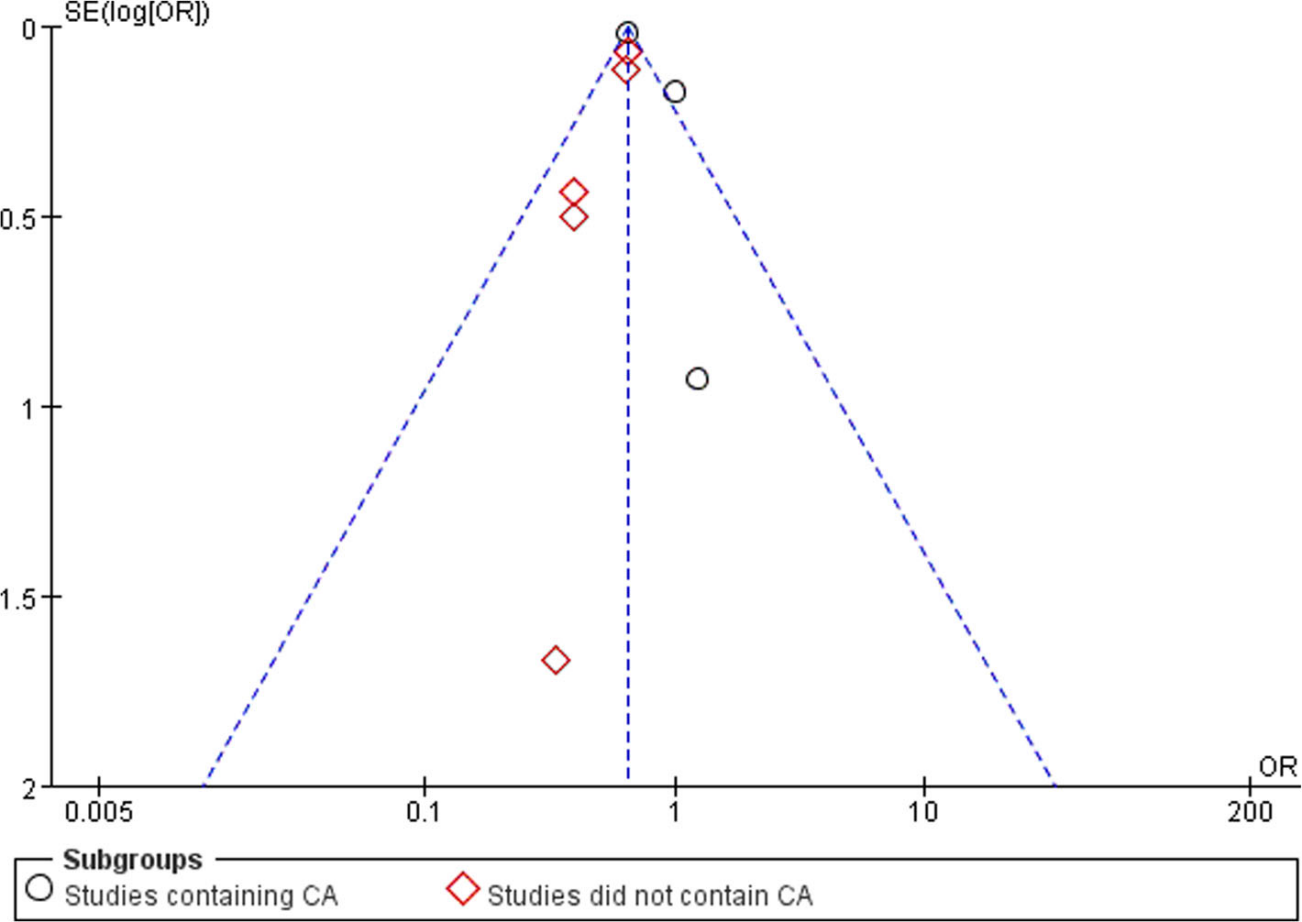
Funnel plot for Subgroup analysis of postoperative complications

The subgroup analysis showed significantly reduced overall complications following laparoscopy in both subgroups (OR,0.65, 95% CI, 0.62 to 0.68; OR,0.64, 95% CI, 0.57 to 0.71). Funnel plot for publication bias showed asymmetry which indicated publication bias may exist (Fig. 5).

### Wound infections

**Fig. 6:**
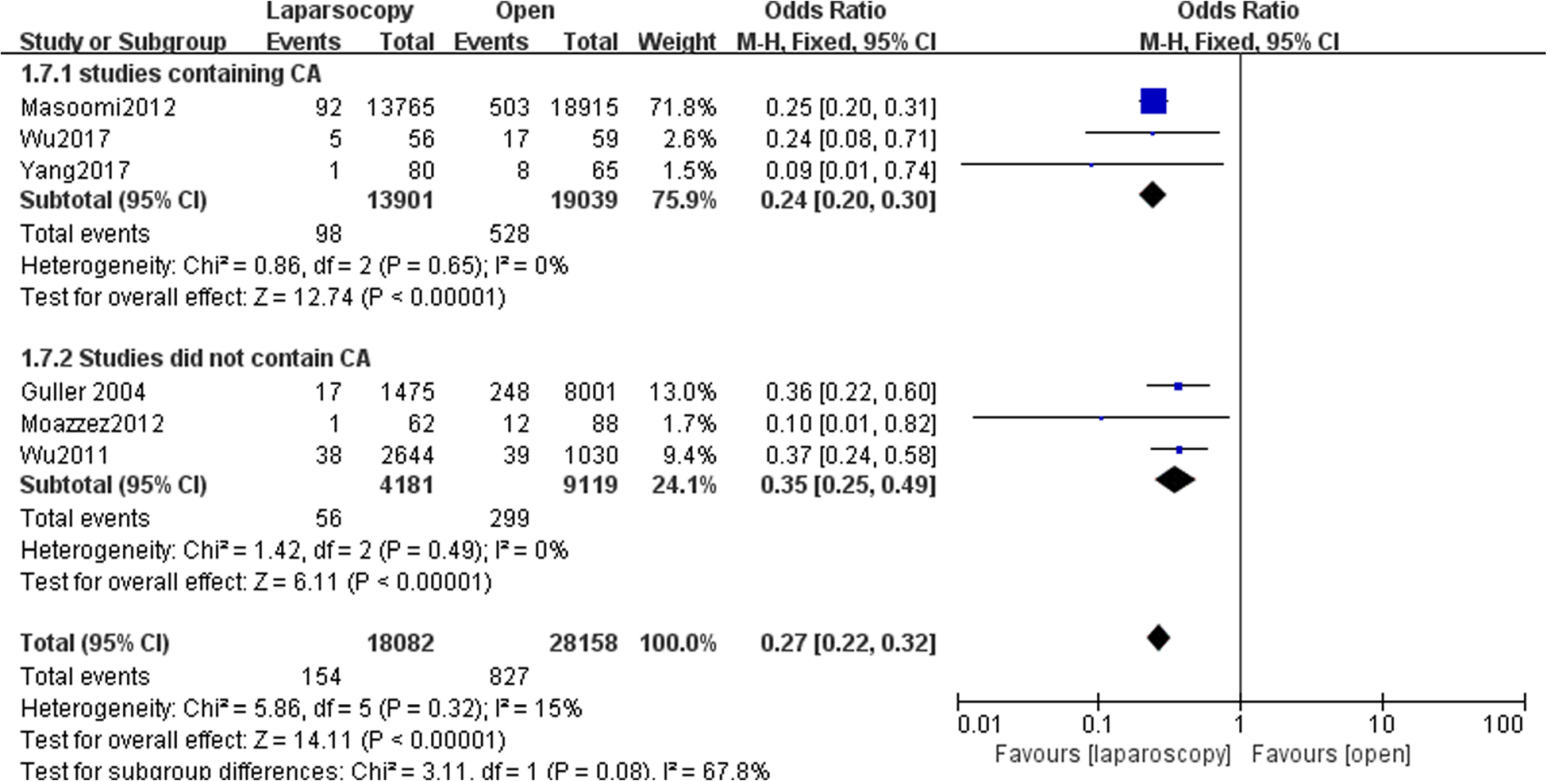
Subgroup analysis of wound infection after laparoscopy and open appendectomy

**Fig. 7:**
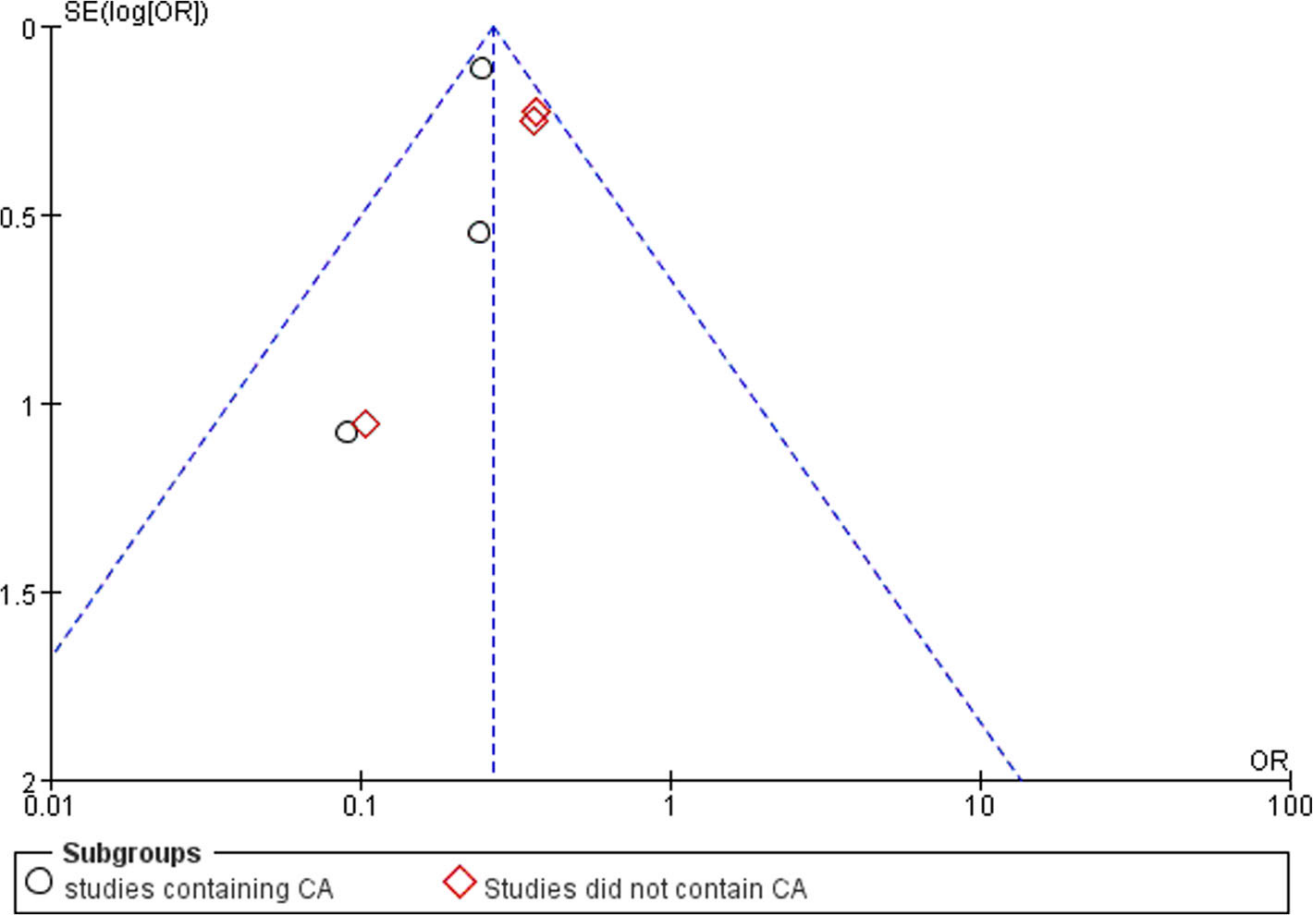
Funnel plot for Subgroup analysis of wound infection

Six studies reported data of wound infection with 18,082 patients in LA group and 28,158 patients in OA group. Wound infection was lower in LA group (OR,0.27, 95% CI, 0.22 to 0.32, shown in Fig. 6). We conducted subgroup analysis. The subgroup analysis showed a reduced rate of wound infection following LA in both subgroups (OR,0.24; 95% CI, 0.20 to 0.30;OR,0.35, 95% CI,0.25 to 0.49). Funnel plot for publication bias showed asymmetry which indicated publication bias may exist (Fig. 7).

### Intra-abdominal abscess

**Fig. 8:**
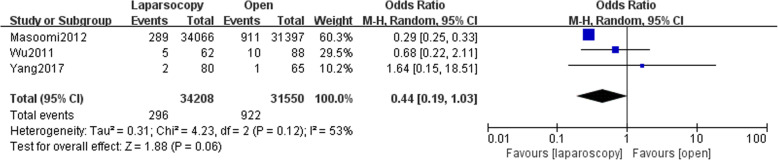
Intra-abdominal abscess after laparoscopy and open appendectomy

**Fig. 9:**
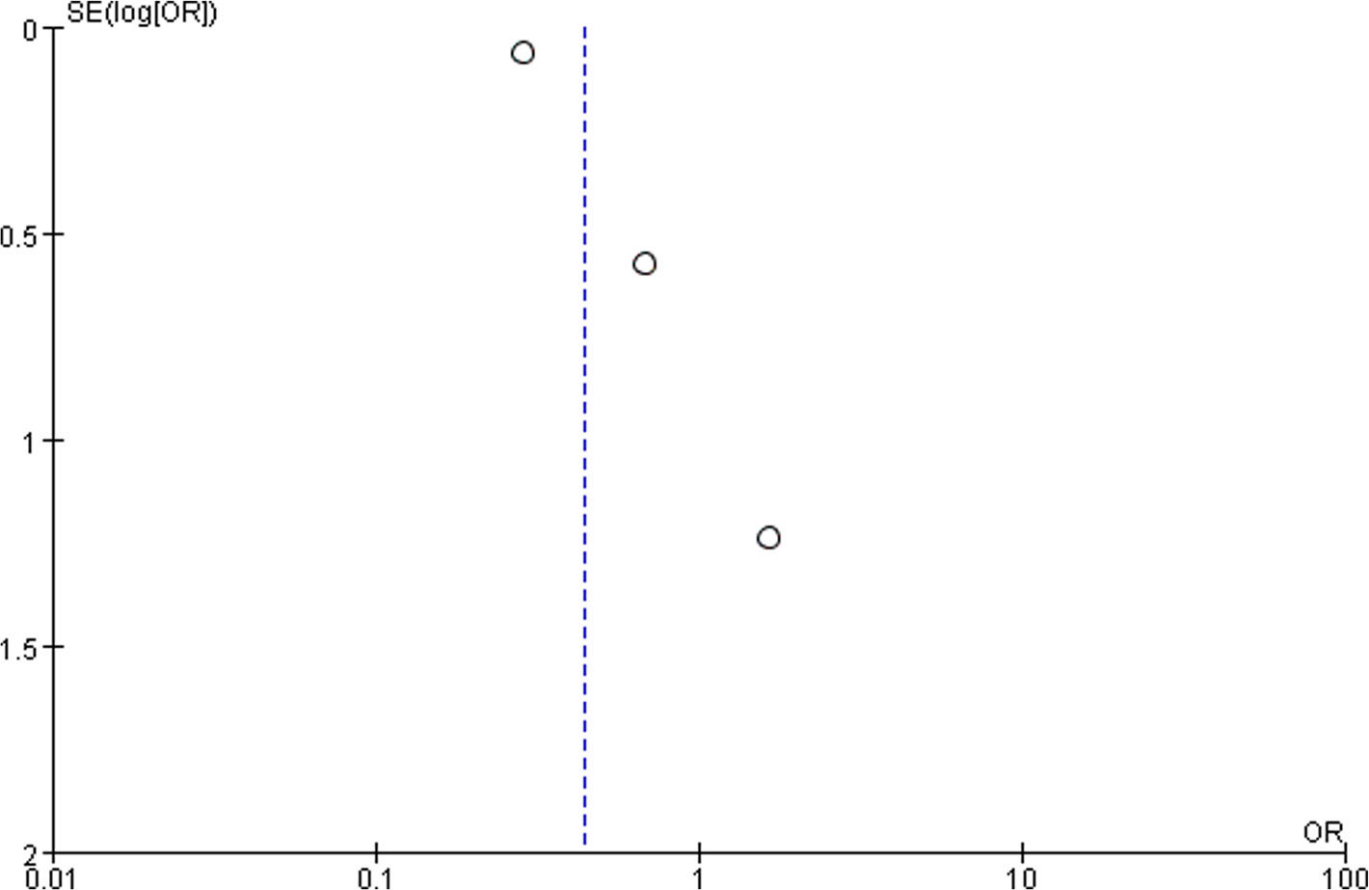
Funnel plot for intra-abdominal abscess

Three studies reported data of postoperative intra-abdominal abscess formation. No significant difference was found between LA and OA (OR,0.44; 95% CI, 0.19 to 1.03, shown in Fig. 8). Medium heterogeneity was found (I^2^ = 53%). Funnel plot for publication bias showed asymmetry which indicated publication bias may exist (Fig. 9).

### Duration of surgery

**Fig. 10:**
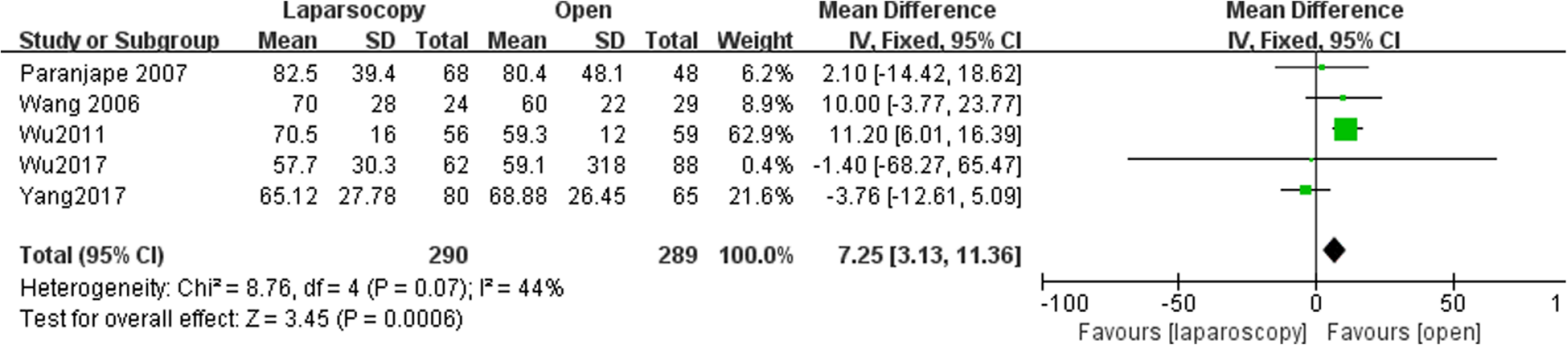
Duration of surgery after laparoscopy and open appendectomy

**Fig. 11:**
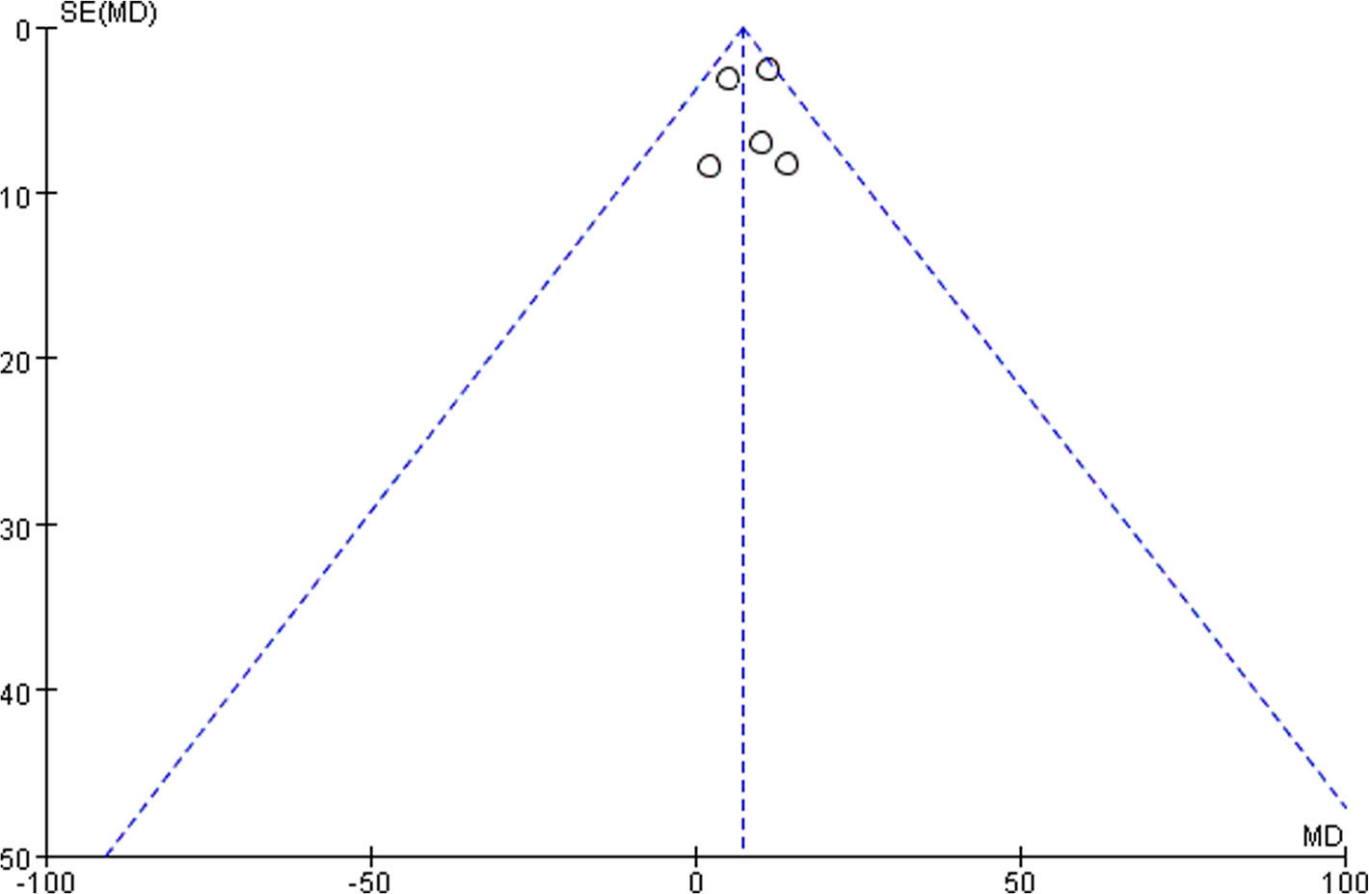
Funnel plot for duration of surgery

Five studies reported data of operating time with 290 patients in LA group and 289 patients in open group. Operating time was longer following LA (MD, 7.25, 95% CI, 3.13 to 11.36, shown in Fig. 10). Moderate heterogeneity was found (I^2^ = 44%). Funnel plot for publication bias detection showed no obvious bias in postoperative mortality (Fig. 11).

### Length of hospital stay

**Fig. 12:**
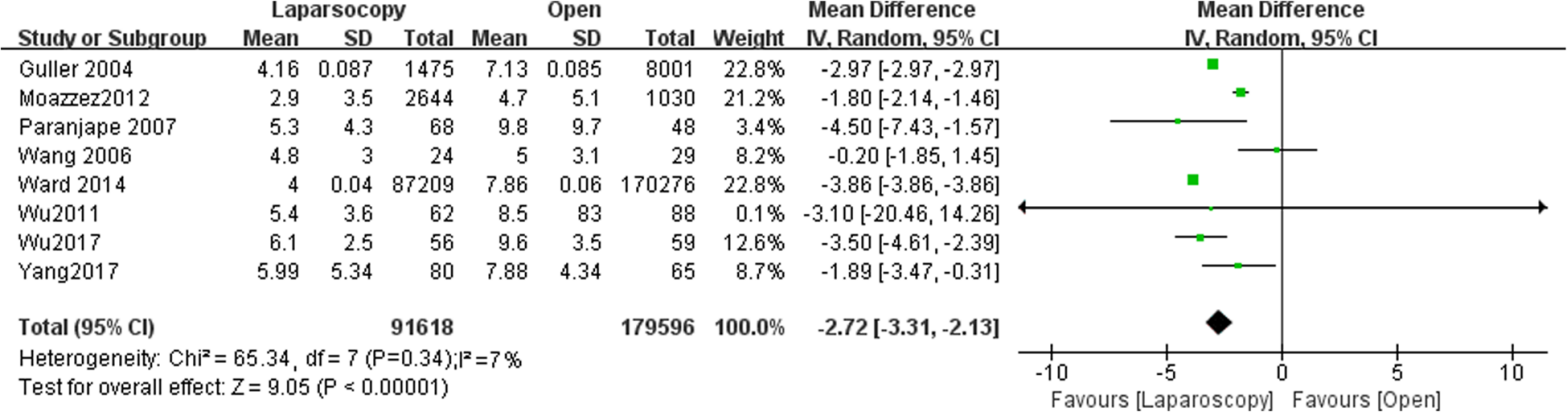
Length of hospital stay after laparoscopy and open appendectomy

**Fig. 13:**
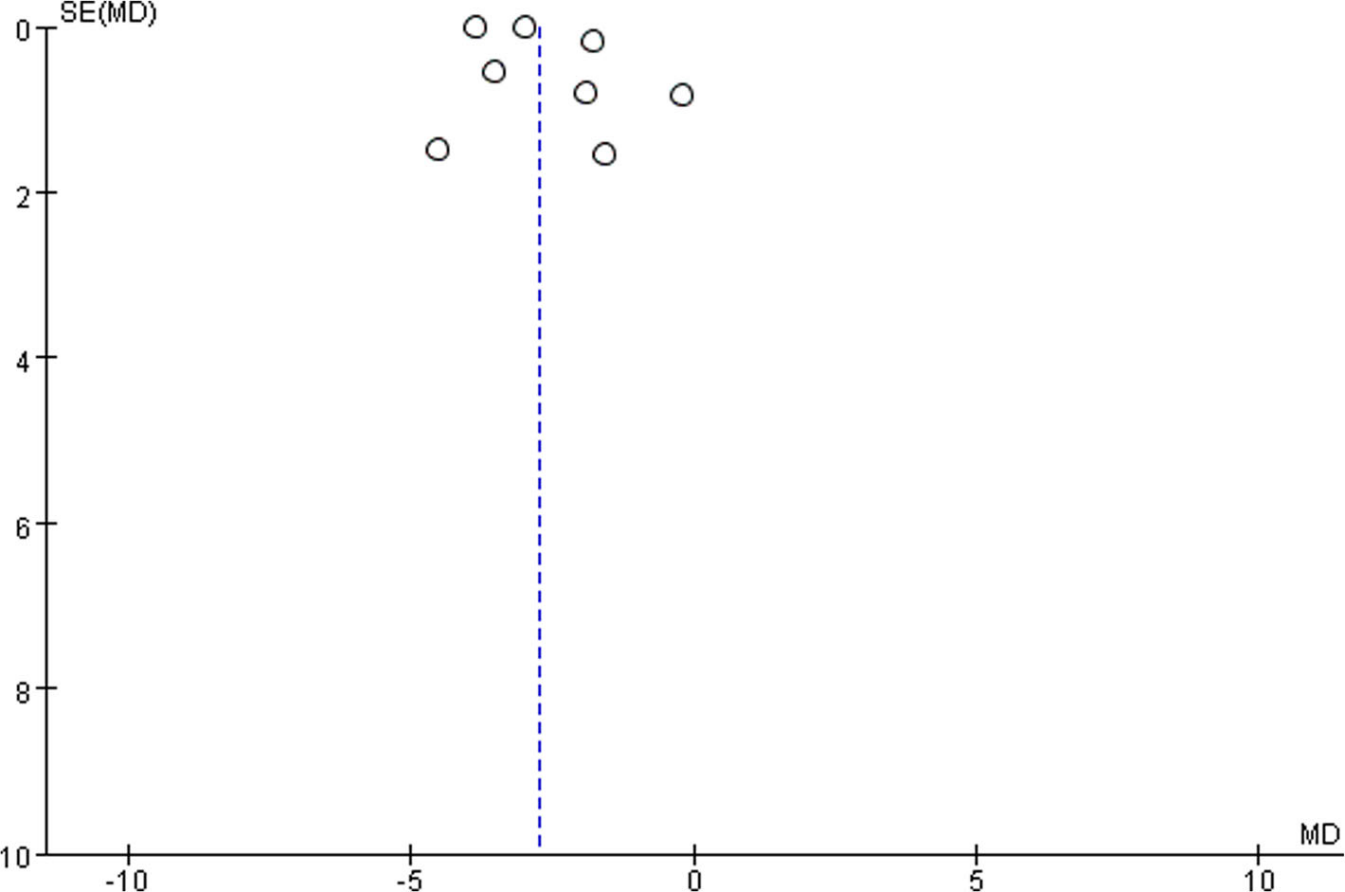
Funnel plot for length of hospital stay

Eight studies have reported length of hospital stay with 91,618 patients in LA group and 179,569 patients in OA group. Length of hospital stay was significantly shorter following LA (MD,-2.72, 95% CI,-3.31 to − 2.13, shown in Fig. 12). Low heterogeneity was found (I^2^ = 7%). Funnel plot for publication bias detection showed no obvious bias in postoperative mortality (Fig. 13).

## Discussion

Elderly patients with appendicitis are associated with higher perforation rate due to atypical symptoms and more comorbidities [27, 28]. Previous studies have demonstrated that postoperative mortality and complication rate were higher in elderly population compared with younger population [29]. The World Society of Emergency Surgery (WSES) have recommended LA for elderly patients in their Jerusalem guidelines for diagnosis and treatment of acute appendicitis [30], however, this recommendation was based on the results from several observational studies without quantitative analysis. As a result, the guideline graded this recommendation as level B, which was generalized from consistent level 2 or 3 (cohort study or case control study) and reflected moderate clinical certainty [31].

Based on our results, the postoperative mortality and complications rate was significantly lower in the LA group. This finding was consistent with adults studies [10]. According to previous studies, death risk of older appendicitis patients is 14 times higher than that of general adult population [32]. With this relatively high risk of mortality, the choice of an appropriate procedure is critical. Laparoscopy seemed to be safer than conventional open procedure due to its low invasiveness and faster recovery. However, the existed studies have also pointed out that in cases of complicated appendicitis, more OA is performed due to more straightforward operating view of the abdominal adhesion and peritonitis. The relatively high postoperative mortality and complication in OA group may be partly accounted for a larger proportion of complicated appendicitis. Perforation rate was also higher in elderly population, due to the atypical symptoms and usually sicker condition of elderly patients, misdiagnosis of a perforated appendix happened in nearly a third of the elderly patients resulting in delay of appropriate treatment [3]. The use of laparoscopy combined with preoperative CT may helped to reduce the rate of misdiagnosis hence prevent perforation. Wound infection was lower following LA in our results. Many studies have demonstrated that compared with OA, LA was associated with less wound infection [33] The use of a wound protective plastic bag when moving out the inflamed appendix in LA may be the primary reason for this result [34]. Less surgical incision, more uncomplicated appendicitis cases in LA group may also account for the lower wound infection rate of LA. The superiority of LA in reducing wound infection was shown in both studies containing CA and uncomplicated appendicitis, existed study suggested that delay of treatment and general condition contributed mostly to wound infection [35]. Our study found no significant difference between LA and OA concerning intra-abdominal abscess, which was inconsistent with previous studies. Limited extracted data pooled in this outcome may be the reason for this finding.

Great heterogeneity is the primary cause to diminish the credibility of a pooled outcome in a meta-analysis [36]. As in the present study, heterogeneity existed in some pooled outcomes. The different postoperative follow up period, the definition of postoperative complication may be the reasons for existed heterogeneity among the studies. Another reason may be the severity of appendicitis in each study, as the range of acute appendicitis covers from uncomplicated appendicitis to abscess and perforation. The precise diagnosis of the type of appendicitis is mainly depended on postoperative pathology, which makes preoperative stratification of the patients unpractical.

Duration of surgery was significantly longer in LA group. Many existed RCTs or meta-analysis focused on adults have also demonstrated the same trend in operating time [37]. Longer operating time can be contributed by several factors, the more equipment used and longer setup time in LA procedure, the learning curve of laparoscopy and the status of the appendix. Length of hospital stay was shorter following LA. This results may due to the less invasiveness of the procedure hence faster recovery. Previous studies have also suggested that after LA, patients can return to normal activity and diet earlier than OA [38]. Although we did not analyze the medical charge between the two groups, many studies have demonstrated that even with higher surgical expenses of LA, the shorter postoperative hospital stay endows the total medical charge almost equivalent between LA and OA [39, 40].

The present study has certain limitations. Firstly, due to the low participant rate of elderly patients in RCTs, all of our enrolled studies were retrospectively observational studies. The results may be influenced by selection bias. Secondly, due to the availability of data from enrolled studies, we only analyzed six outcomes. More comparable outcomes like postoperative pain, return to normal activity and readmission were not investigated. Thirdly, some of the enrolled studies included both uncomplicated and complicated appendicitis, the type of appendicitis and comorbidities of the patient can be confounding factors for the results. Fourthly, although we tried to search as comprehensively as possible to avoid publication bias, it was still detected in overall complications, wound infection and intra-abdominal abscess. The results might be exaggerated due to publication bias. Fifth, study by Masoomi et al. and studies by Kim et al. and Moazzez et al. which analyzed data from the National Inpatient sample database (NIS) and National Surgical Quality Improvement Program (NSQIP) may have data overlap. These two database are both primarily composed by hospitals from the United States hence double count of patients may exist in the present meta-analysis.

The current meta-analysis showed that LA is a safe and feasible procedure for elderly appendicitis patients with lower rate of postoperative mortality and complication and shorter hospital stay. Laparoscopy should be recommended to elderly patients when there are no contraindications. However, larger high quality RCTs are still needed to form a more solid conclusion.

## Conclusions

For elderly patients with appendicitis, laparoscopy is associated with less postoperative mortality and complication, less wound infection, shorter hospital stay. The use of laparoscopy is safe and feasible for elderly population.

## Additional files

Additional file 1: Searching Terms in MEDLINE. (DOCX 12 kb)

Additional file 2: NOS scale for enrolled studies. (DOCX 15 kb)

## Abbreviations

CI: Confidence intervals
LA: Laparoscopic appendectomy
MD: Mean difference
NOS Scale: Newcastle Ottawa Quality assessment scale
OA: Open appendectomy
OR: Odds ratio
RCT: Randomized control studies
WSES: World society of emergency surgery
