# Supplementary material for: Laparoscopy versus open appendectomy for elderly patients, a meta-analysis and systematic review
Source: BMC Surg. 2019 May 28;19:54. doi: 10.1186/s12893-019-0515-7 (PMC6540400; doi:10.1186/s12893-019-0515-7)
Supplement: Supplementary file 1 — Searching Terms in MEDLINE. (DOCX 12 kb) [file 12893_2019_515_MOESM1_ESM.docx]

Searching Terms in MEDLINE

#1 Appendicitis"[Mesh]

#2 Ruptured Appendicitis[Title/Abstract] OR Appendicitis, Ruptured[Title/Abstract] OR Perforated Appendicitis[Title/Abstract] OR Appendicitis, Perforated[Title/Abstract]

#3 #1 OR #2

#4 Geriatrics"[Mesh]

#5 Gerontology[Title/Abstract] OR elderly[Title/Abstract] OR aged[Title/Abstract] OR older[Title/Abstract]

#6 #4 OR #5

#7 #3 AND #6

Searching Terms in Embase

#1 appendicitis'/exp

#2 'geriatrics'/exp

#3 elderly OR aged OR older:ab,ti

#4 #2 OR #3

#5 #1 AND #4

Searching Terms in Cochrane

#1 MeSH descriptor: [Appendicitis] explode all trees

#2 MeSH descriptor: [Appendectomy] explode all trees

#3 MeSH descriptor: [Geriatrics] explode all trees

#4 (#1 or #2 ) AND (#3 OR elderly OR old OR aged)
