# Supplementary material for: Laparoscopy versus open appendectomy for elderly patients, a meta-analysis and systematic review
Source: BMC Surg. 2019 May 28;19:54. doi: 10.1186/s12893-019-0515-7 (PMC6540400; doi:10.1186/s12893-019-0515-7)
Supplement: Supplementary file 2 — NOS scale for enrolled studies. (DOCX 15 kb) [file 12893_2019_515_MOESM2_ESM.docx]

Supplementary 2 NOS scale for enrolled studies

|  | Selection | | | | Comparability | | Outcome assessment | | | Score |
| --- | --- | --- | --- | --- | --- | --- | --- | --- | --- | --- |
| Study | 1 | 2 | 3 | 4 | 5 | 6 | 7 | 8 | 9 |  |
| Guller | * | * | * | * | * | * | * | * |  | 8 |
| Harrell | * | * | * | * | * | * | * | * |  | 8 |
| Wang | * | * | * | * | * | * | * | * | * | 9 |
| Paranjape | * | * | * | * | * |  | * | * |  | 7 |
| Kim | * | * | * | * | * | * | * | * |  | 8 |
| Wu | * | * | * | * | * | * | * | * |  | 8 |
| Masoomi | * | * | * | * | * |  | * | * |  | 7 |
| Farrerese | * | * | * | * | * | * | * | * |  | 8 |
| Moazzez | * | * | * | * | * | * | * | * |  | 8 |
| Ward | * | * | * | * | * | * | * | * |  | 8 |
| Wu | * | * | * | * | * | * | * | * |  | 8 |
| Yang | * | * | * | * | * |  | * | * |  | 7 |
|  |  |  |  |  |  |  |  |  |  |  |
